# *Mycobacterium tuberculosis* subverts macrophage-mediated defense through exploitation of the Zn transporter ZIP8

**DOI:** 10.1016/j.isci.2026.116276

**Published:** 2026-06-09

**Authors:** Eusondia Arnett, Miranda Lumbreras, Elizabeth Hernandez, Daniela Campos, Chrissy M. Leopold Wager, Susanta Pahari, Dahlia Matouba, Brenden F. Determann, Charles Renshaw, Brandon Longstreet, Olga Gonzalez, Vinay Shivanna, Edward J. Dick, Abul Azad, Charlie Pyle, Deandra Smith, Vartika Tomar, Evelyn Guirado, Anil K. Ojha, Joanna Melia, Daren Knoell, Larry S. Schlesinger

**Affiliations:** 1 Host Pathogen Interactions, Texas Biomedical Research Institute, San Antonio, TX 78227, USA; 2 Duke University School of Medicine, Durham, NC 27710, USA; 3 Department of Microbiology, Immunology and Molecular Genetics, UT San Antonio Health Science Center, San Antonio, TX 78229, USA; 4 Southwest National Primate Research Center, Texas Biomedical Research Institute, San Antonio, TX 78227, USA; 5 University of Nebraska Medical Center, Omaha, NE 68198, USA; 6 HIPRA, Girona, Spain; 7 Division of Genetics, Wadsworth Center, New York State Department of Health, Albany, NY 12210, USA; 8 Division of Gastroenterology and Hepatology, Department of Medicine, Johns Hopkins University School of Medicine, Baltimore, MD 21218, USA

**Keywords:** molecular biology, immunity, microbiology

## Abstract

Zinc (Zn) is an essential micronutrient whose concentration and location are tightly regulated by Zn transporters. *Mycobacterium tuberculosis (M.tb)* resides in macrophage phagosomes, where it requires access to micronutrients, including Zn. Our data show that ZIP8 is the only Zn transporter in human macrophages highly induced by *M.tb* and is enriched at the *M.tb* phagosome. Using human and murine macrophages and mice deficient in ZIP8, we found that *M.tb* exploits ZIP8 to enhance its growth. ZIP8 imports Zn into the cytosol and out of the phagosome to subvert Zn poisoning. Cytosolic Zn dampens NF-κB activation and pro-inflammatory cytokine production while enhancing matrix metalloproteinase (MMP) production. Understanding the Zn- and ZIP8-dependent host response to *M.tb* infection is critical since dietary Zn deficiency and polymorphic ZIP8 variants are associated with increased susceptibility to tuberculosis and other infectious diseases.

## Highlights

• ZIP8 colocalizes with the *Mycobacterium tuberculosis* phagosome in human macrophages
• ZIP8 imports Zn into macrophages and out of *Mycobacterium tuberculosis* phagosomes
• ZIP8 reduces NF-κB activity and induces matrix metalloproteinase expression
• ZIP8 is important for *Mycobacterium tuberculosis* growth

## Graphical abstract

**Figure:**
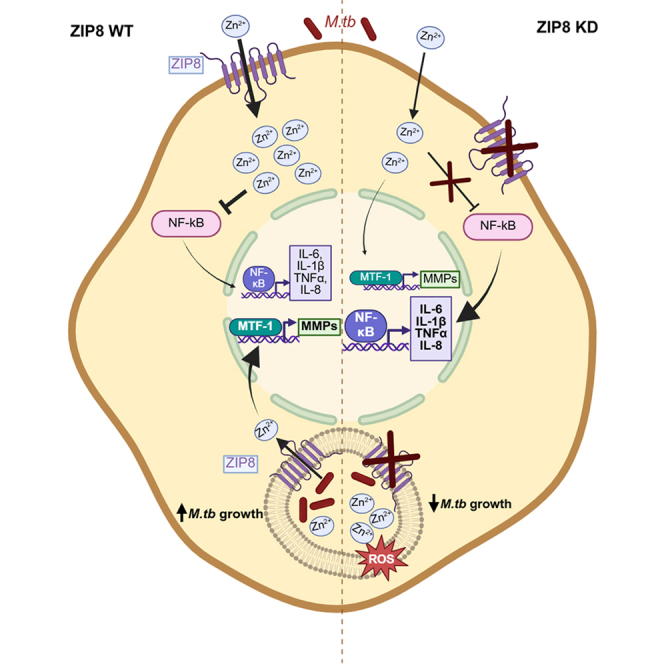

## Introduction

Tuberculosis (TB) is a leading cause of death due to infectious diseases. TB diagnosis and treatment were severely disrupted during the worst years of the COVID-19 pandemic, with global TB incidence rate increasing by 4.6% from 2020 to 2023, in stark contrast to the 2% annual reductions seen between 2010 and 2020. In 2023 and 2024, there were over 10 million new TB cases and 1.2 million deaths each year, which is second only to COVID-19 in the past three years.^1^^,^^2^ A number of factors have been implicated in increased susceptibility to TB, including HIV infection, smoking, diabetes, and malnutrition.^1^ New approaches to effective therapeutic and vaccine interventions require a more thorough understanding of infection pathogenesis.

Zinc (Zn) is the second most prevalent trace metal in vertebrates and is essential for cell survival and healthy immunity.^3^ Zn deficiency, associated with reduced Zn intake, affects 2 billion people worldwide and is implicated in susceptibility to infectious diseases, including COVID-19 and TB.^3^^,^^4^^,^^5^ Zn is also essential for prokaryotic cells, thereby creating a “tug of war” for nutrient acquisition between host and microbe. In nutritional immunity, the host sequesters metals away from pathogens to restrict growth, thereby inducing specific metal starvation responses in the pathogen, including *Mycobacterium tuberculosis* (*M.tb)*, which responds to Zn starvation by remodeling and hibernating the ribosome.^6^^,^^7^ In contrast, the host can also divert high metal concentrations to kill pathogens through increased oxidative stress and protein function disruption.^8^^,^^9^ *M.tb* is a highly adept intracellular bacterium, establishing infection through residence within distinctive macrophage phagosomes.^10^ The capacity of *M.tb* to persevere in the phagosome depends in part on access to micronutrients. Zn accumulates in the *M.tb* phagosome^11^^,^^12^ and may be toxic to *M.tb*. To combat this, *M.tb* and *Mycobacterium marinum* induce the expression of the heavy metal efflux P_1_-type ATPase CtpC in response to Zn to export Zn out of the bacterium and protect against Zn toxicity.^11^^,^^13^

Zn homeostasis and function are regulated by ten Zn export proteins (ZnTs), also known as solute carrier family proteins (SLC30A1-10), which export Zn from the cytoplasm, and fourteen Zn import, Zrt-Irt-like-proteins (ZIPs; SLC39A1-14), which import Zn into the cytoplasm from extracellular and intracellular stores such as the phagosome.^14^^,^^15^ Among the 24 Zn transporters, ZIP8 is the only one highly induced in response to bacteria in myeloid and epithelial cells and regulates host defense in key immune cells.^3^^,^^16^ The Zn transporter ZIP8 was first discovered following its induction in monocytes in response to *M. bovis* BCG infection.^17^ Since then, we have shown that TNFα and LPS also increase ZIP8 expression.^4^^,^^18^^,^^19^ ZIP8 is ubiquitously expressed, with the highest expression in the kidney and lungs.^3^ Upon bacterial challenge and depending on cell type, ZIP8 can translocate to plasma, endosomal, or lysosomal membranes and transport Zn into the cytosol.^20^^,^^21^ ZIP8 is a highly polymorphic gene whereby defective alleles alter host function in inflammatory conditions and *S. aureus* infection.^22^^,^^23^^,^^24^^,^^25^^,^^26^^,^^27^ A frequently occurring ZIP8 variant dysfunctional allele (rs13107325; Ala391Thr risk allele) is strongly associated with inflammation-based disorders and bacterial infection.^3^

Our group has discovered that ZIP8 is constitutively present at low levels in human macrophages and uniquely induced upon *M.tb* infection relative to all other ZnT and ZIP transporters.^4^ However, the role of ZIPs, including ZIP8, in human macrophages during *M.tb* infection remains unknown. Here, we queried the impact of Zn and ZIP8 on *M.tb* infection, using primary human and murine macrophages (the predominate cell niche for *M.tb* during infection), the latter from myeloid-specific ZIP8 knockout (KO) mice^28^^,^^29^ and a mouse model that mimics the human ZIP8 A391T loss of function variant allele.^16^ We show that ZIP8 rapidly co-localizes to the *M.tb* phagosome within 10 min, and this is maintained out to at least 48 h. In doing so, ZIP8 transports Zn out of the *M.tb* phagosome and into the cytosol, decreasing phagosome Zn concentrations, which curtails host-mediated toxicity directed at *M.tb*. Mobilization of Zn into the cytosol inhibits NF-κB activity and dampens pro-inflammatory cytokine expression and release during *M.tb* infection of human macrophages and mice. In addition, ZIP8-mediated re-localization of Zn to the cytosol drives the expression of Metal Regulatory Transcription Factor 1 (MTF-1) that subsequently induces the production of matrix metalloproteinases (MMPs). The culmination of ZIP8-driven change in these activities leads to increased *M.tb* growth in macrophages and dissemination in mice. Together, these data advance our fundamental understanding of mechanisms underlying Zn and ZIP8’s role in regulating host immune responses to *M.tb*.

## Results

### ZIP8 rapidly co-localizes with *M.tb* in the phagosome

**Figure 1:**
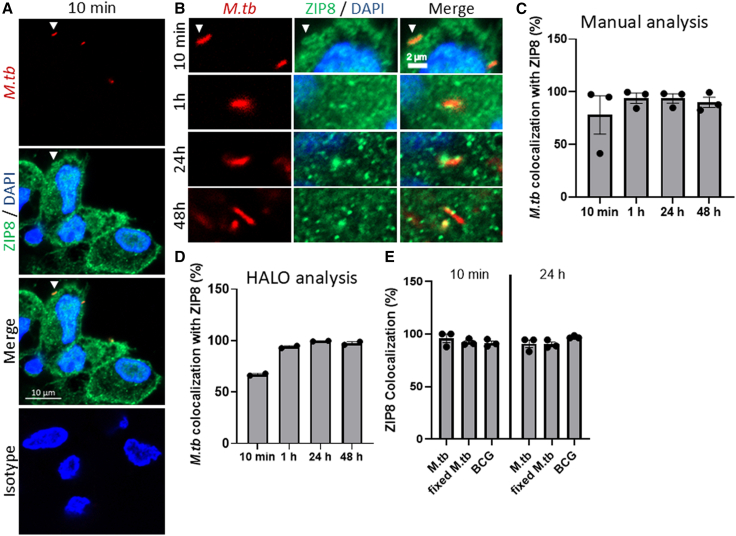
ZIP8 co-localizes with *M.tb* in macrophages (A–D) MDMs were infected with mCherry *M.tb* at MOI 5 and fixed at the indicated times. MDMs were permeabilized, then ZIP8 (green), and the nuclei (blue) were stained. (A) Representative images acquired at 63×, the white arrowhead indicates one example of *M.tb* colocalization with ZIP8, scale bar, 10 μm. (B) Zoomed-in images at each time point, the white arrowhead indicates one example of *M.tb* colocalization with ZIP8, scale bar, 2 μm. (C) ZIP8 colocalization with *M.tb* was quantified manually, by counting at least 50 *M.tb* per experiment. Results are mean ± SEM (*n* = 3 independent biological replicates with 2 technical replicates each). Each dot indicates an independent biological replicate. (D) ZIP8 colocalization with *M.tb* was quantified with HALO software. Results are mean ± SEM (*n* = 2 independent biological replicates with 2 technical replicates each). Each dot indicates an independent biological replicate. (E) MDMs were infected with mCherry *M.tb* (viable or fixed) or viable mCherry BCG for the indicated times, then fixed and ZIP8 stained as in (A). ZIP8 colocalization with *M.tb* or BCG was quantified manually as described for (C). Results are mean ± SEM (*n* = 3 independent biological replicates with 2 technical replicates each). Each dot indicates an independent biological replicate.

We have previously shown that macrophages constitutively produce ZIP8, and that ZIP8 is unique among the other Zn transporters as the most responsive to *M.tb* infection in both primary human monocyte-derived macrophages (MDMs) and human alveolar macrophages (HAMs).^4^ ZIP8 localizes to plasma, endosomal, and lysosomal membranes.^20^^,^^21^^,^^30^^,^^31^^,^^32^ Our prior work showed that in primary human macrophages, ZIP8 co-localizes with *M.tb* and exhibits some co-localization with early endosomes (i.e., transferrin receptor, which transports iron), but limited co-localization with late endosomes/lysosomes (LAMP-1), after 48 h of infection.^4^ Here, we assessed *M.tb/*ZIP8 co-localization over a time course to determine how rapidly co-localization occurs. We observed ZIP8 present throughout the cells, with the increased co-localization of ZIP8 with the phagosome membrane around *M.tb* as early as 10 min following infection (78 ± 18% co-localization, Figures 1A–1C). *M.tb* co-localization increased to 93 ± 5% by 1 h and was maintained through at least 48 h of infection (Figures 1B and 1C). To confirm our results, we employed an independent assessment using HALO software to enumerate the number of fluorescent bacteria that have overlapping pixels with ZIP8 signal. This approach confirmed that *M.tb* rapidly co-localizes with ZIP8, and the majority of *M.tb* (93 ± 2%) in the phagosome co-localizes with ZIP8 by 1 h (Figure 1D). We next investigated whether ZIP8 colocalizes with other mycobacteria, and whether *M.tb* actively recruits and/or retains ZIP8 at the phagosome. We infected MDMs with non-viable *M.tb* (following fixation, which allows *M.tb* to retain cell wall architecture^33^) or live BCG (which is attenuated and does not produce the *M.tb* virulence factor ESX-1, among others). Both dead *M.tb* and live BCG co-localize with ZIP8 to a similar extent as live *M.tb* after just 10 min. Similar to live *M.tb*, this was maintained through at least 24 h (Figure 1E). This result indicates that ZIP8 co-localization is not actively induced by live *M.tb*.

### ZIP8 contributes to Zn import into the cytosol and Zn export from the phagosome

**Figure 2:**
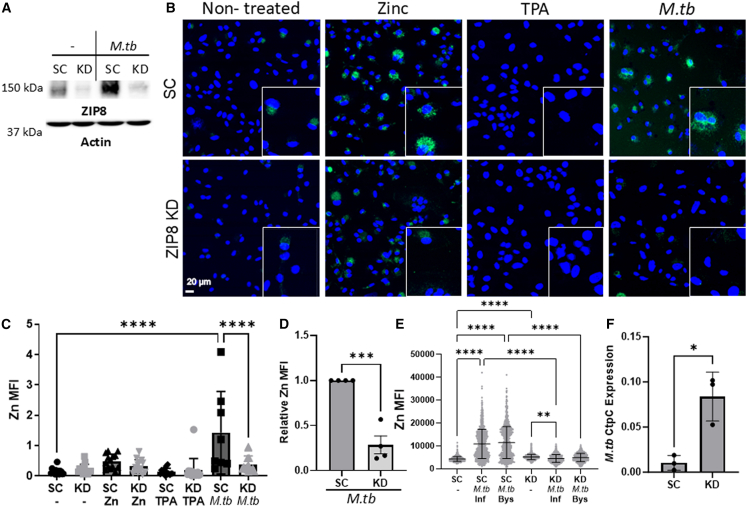
ZIP8 contributes to Zn import into the macrophage cytosol and Zn export out of the phagosome (A–D) MDMs were transfected with scrambled control (SC) or ZIP8-specific (KD) siRNA, then treated with zinc (18 μM, 2 h), TPA (1 μM, 30min), or *M.tb* (MOI 10, 2 h). (A) After 48 h, cells were lysed to assess ZIP8 knockdown efficiency by western blot (mean 71 ± 3%). See Figure S7 for uncropped blots. (B) Cells were fixed, then stained with FluoZin-3 (Zn, green) and DAPI (blue). Representative images, scale bar, 20 μm. (C and D) Mean fluorescent intensity (MFI) of FluoZin-3 in 1,000 macrophages per experiment was automatically quantified with FIJI. (C) Results are mean ± SD, of at least 8 different images, representative of 4 independent biological replicates with 2 technical replicates each. Each point is one image containing >40 MDMs, One-way ANOVA with Tukey’s post-test, ∗∗∗∗*p* < 0.0001. (D) FluoZin-3 MFI during *M.tb* infection was quantified, results are relative to *M.tb* scrambled control, mean ± SEM (*n* = 4 independent biological replicates with 2 technical replicates each), unpaired *t* test, ∗∗∗*p* < 0.001. Each dot indicates an independent biological replicate. (E) MDMs were transfected with scrambled control (SC) or ZIP8-specific (KD) siRNA, then infected with mCherry *M.tb* (MOI 10, 2 h). FluoZin-3 MFI was quantified in over 500 different MDMs per condition, in uninfected cells or following infection in infected (Inf, mCherry+) or bystander (Bys, mCherry-) MDMs. Mean ± SD, each dot indicates one MDM, one-way ANOVA with Tukey’s post-test, ∗∗*p* < 0.01 and ∗∗∗∗*p* < 0.0001. Representative of 3 independent biological replicates with 2 technical replicates each. (F) MDMs were transfected with scrambled control (SC) or ZIP8 (KD) siRNA, then infected with *M.tb* (MOI 10) for 24 h. RNA was isolated, and *M.tb* CtpC expression was assessed by qRT-PCR. Results are mean ± SD of triplicate technical replicates, representative of three independent biological replicates, unpaired *t* test, ∗*p* < 0.05. Each dot indicates a technical replicate.

We next determined whether ZIP8 regulates intracellular Zn levels during *M.tb* infection. Human macrophages were transfected with ZIP8 or scrambled control siRNA, which resulted in 71 ± 3% ZIP8 knockdown (Figure 2A). *M.tb-*driven ZIP8 induction in human macrophages results in a glycosylated protein identified as a broad band by western blot.^4^ siRNA-transfected macrophages were infected with *M.tb* or treated with Zn or tris(2-pyridylmethyl)amine (TPA, an intracellular Zn chelator) as positive and negative controls, respectively, for assessment by a fluorescent Zn probe. Macrophages were then fixed and stained with the Zn-specific fluorescent probe FluoZin-3. As expected, we observed increased staining with Zn treatment, which was reduced with TPA or ZIP8 knockdown (Figures 2B and 2C). Notably, *M.tb* infection increased intracellular Zn levels, and this was significantly reduced by 71.5 ± 0.1% when ZIP8 was knocked down (Figures 2B–2D). We next determined whether the increased Zn observed during *M.tb* infection was specific to *M.tb-*infected macrophages. We infected MDMs with mCherry fluorescent *M.tb*, and assessed Zn levels in both infected and bystander macrophages. *M.tb* infection led to increased Zn levels in both infected and bystander macrophages, in a ZIP8-dependent manner (Figure 2E). Thus, we conclude that *M.tb* infection of macrophages via ZIP8 leads to a general increase in Zn import into human macrophages, suggestive of a paracrine effect.

We next assessed if ZIP8 imports Zn into the cytosol from intracellular stores, including the phagosome (and possibly the extracellular space), thereby potentially reducing phagosomal Zn levels. We utilized a three-pronged approach to assess this: 1) using the fluorescent probe FluoZin-3 to visualize Zn in the phagosome-lysosome pathway, 2) using a Zn reporter *M.tb* strain,^6^ and 3) using qRT-PCR to assess *M.tb* Zn-responsive gene expression in the phagosome. We were unable to distinguish phagosomal from cytosolic Zn levels with the fluorescent probe FluoZin-3, in line with a previous study reporting that FluoZin-3 was unable to detect phagosomal Zn in the absence of additional stimulation.^34^ We then pivoted to fluorescent imaging of a *M.tb* Zn reporter strain that constitutively expresses mCherry and produces the green fluorescent protein Dendra2 under Zn limiting conditions (under control of the *mtb*_*C*_*-* promoter, which is important for ribosome hibernation and is turned on when Zn is limiting)^6^ (Figure S1A). Thus, the Dendra2 signal is inversely correlated with Zn levels (Figures S1A and S1B). We infected MDMs with this reporter strain and used live-cell imaging to visualize the change in the frequency of Dendra2-expressing *M.tb* in phagosomes over time. We observed a progressive reduction in the number of Dendra2-positive *M.tb* except in the case of cells from one donor (Figures S1C and S1D), thereby indicating increased phagosomal Zn levels over time. Not only did the number of Dendra2-positive *M.tb* decrease, but the Dendra2 fluorescent signal also decreased over time; there was a 54.0 ± 0.06% reduction in the ratio of Dendra2 to mCherry fluorescence (Figure S1E). This supports previous work indicating that Zn levels increase between 1 and 24 h of infection in murine macrophages and *in vivo* in mice.^6^^,^^12^ Here, we show that Zn levels start to increase as early as 12–16 h post-infection and continue to increase until about 60 h post-infection, when they plateau for at least another 8 h in human macrophages. However, we did not observe a consistent change in Dendra2-positive *M.tb* or changes in Dendra2/mCherry signal in response to ZIP8 KD, likely indicating that the magnitude of Zn changes following ZIP8 KD is insufficient to impact the responsiveness of the reporter strain.

As a third approach to assess changes in phagosomal Zn levels, we probed *M.tb* gene expression in the phagosome. We focused specifically on *M.tb* gene expression since the expression of host Zn-regulated genes would indicate altered Zn levels in the cytosol or nucleus, not the phagosome. Upon Zn exposure *M.tb* upregulates expression of the heavy metal efflux P_1_-type ATPase CtpC, and thus changes in CtpC expression can be used to indicate changing Zn levels in the *M.tb* phagosome.^11^^,^^34^ We isolated RNA from *M.tb*-infected macrophages,^35^ and performed qRT-PCR to determine the relative level of CtpC expression in *M.tb* recovered from ZIP8 KD and control MDMs. A mean 30-fold increase in CtpC expression was observed in ZIP8 KD macrophages (Figure 2F), indicating increased phagosomal Zn with ZIP8 KD. These data are consistent with a role for ZIP8 in transporting Zn out of the *M.tb* phagosome, which we posit mitigates Zn poisoning and thereby enables *M.tb* survival. Altogether, these data indicate that ZIP8 plays an important role in regulating macrophage intracellular Zn levels, increasing overall intracellular Zn while reducing Zn specifically in the *M.tb* phagosome.

### ZIP8 inhibits NF-κB and dampens cytokine release during *M.tb* infection

**Figure 3:**
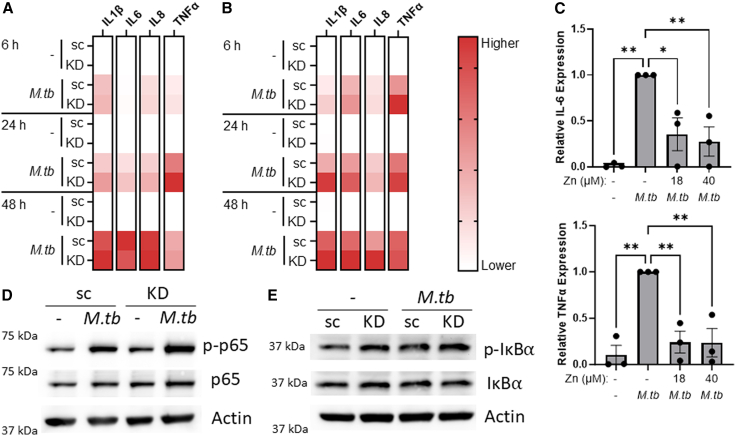
ZIP8 dampens pro-inflammatory cytokine release and NF-κB activation during *M.tb* infection of macrophages (A and B) MDMs transfected with scrambled control (sc) or ZIP8 (KD) siRNA were infected with *M.tb* (MOI 5) for 6-48 h. (A) RNA was collected, and mRNA expression was assessed by qRT-PCR. Results are mean *n* = 3 (IL-8: 6,24,48 h; IL-1β, IL-6, TNFα: 24 h) or 4 (IL-1β, IL-6, TNFα: 6 and 48 h) independent biological replicates with 3 technical replicates each. See Figure S2 for representative data. (B) Supernatant was collected, and cytokine release was assessed by ELISA. Results are mean of *n* = 3 (24 h), or 4 (6, 48 h) independent biological replicates with 3 technical replicates each. See Figure S2 for representative data. (C) MDMs were infected with *M.tb* (MOI 5) for 2 h, washed, and incubated in the presence or absence of the indicated concentration of Zn for 48h. RNA was collected, and mRNA expression was assessed by qRT-PCR. Results are mean ± SEM (*n* = 3 independent biological replicates with 3 technical replicates each). Each dot indicates an independent biological replicate. One-way ANOVA with Dunnett’s post-test, ∗*p* < 0.05 and ∗∗*p* < 0.01. (D and E) MDMs transfected with scrambled control (sc) or ZIP8 (KD) siRNA were infected with *M.tb* (MOI 10, synchronized phagocytosis) and protein lysates collected after 10min (D) or 2min (E) for WB. Representative of six (D) or two (E) independent biological replicates. Densitometry results are in Figure S3, uncropped blots in Figure S7.

ZIP8-mediated transport of Zn into the cytosol regulates the expression of several pro-inflammatory cytokines following LPS stimulation in other cell types,^36^ but whether this occurs in primary human macrophages, and during *M.tb* infection, is unknown. To assess this, MDMs were transfected with ZIP8 or scrambled control siRNA, then infected with *M.tb*. Cytokine gene expression and protein release were assessed by qRT-PCR and ELISA, respectively, over time. *M.tb* infection induced gene expression and release of IL-6, IL-1β, TNFα, and IL-8 in a time-dependent manner, and this was further increased with ZIP8 knockdown (Figures 3A, 3B, S2A, and S2B). TNFα expression and release were primarily regulated by ZIP8 early during infection (6 and 24 h), while IL-1β, IL-6, and IL-8 were primarily regulated at 6–48 h during infection (Figures 3A, 3B, S2A, and S2B). This is likely due to ZIP8-mediated Zn transport, since the addition of Zn at physiologically relevant Zn concentrations (18 μM)^5^ reduced cytokine expression (Figure 3C) without causing macrophage toxicity (Figures S2C and S2D).

Given NF-κB’s central role in regulating cytokine responses, we next determined whether the presence or absence of ZIP8 modifies the extent of NF-κB component phosphorylation as a standard proxy for activity during *M.tb* infection of macrophages. We observed increased p65 phosphorylation with *M.tb* infection, which was moderately increased with ZIP8 KD (Figure 3D). This was observed after just 10 min following synchronized phagocytosis and maintained through at least 30 min (Figures 3D and S3A). NF-κB is negatively regulated through direct interaction with the IκBα complex, which under unstimulated conditions binds to and inhibits NF-κB phosphorylation and nuclear translocation. Following stimulation, however, IκBα is phosphorylated, which leads to IκBα degradation, releasing NF-κB components, including p65, which is then phosphorylated and translocates to the nucleus to drive gene expression. Since we observed changes in p65 phosphorylation within 10 min of infection, we assessed IκBα phosphorylation at an earlier time point. Within 2 min, we observed increased IκBα phosphorylation with ZIP8 KD and *M.tb* infection (Figures 3E and S3B). Altogether, our data indicate that Zn import into the cytosol in human macrophages through ZIP8 dampens IκBα phosphorylation, thereby limiting NF-κB activation and expression and release of pro-inflammatory cytokines normally associated with *M.tb* control.

### ZIP8 increases MTF-1 and MMP expression during *M.tb* infection of macrophages

**Figure 4:**
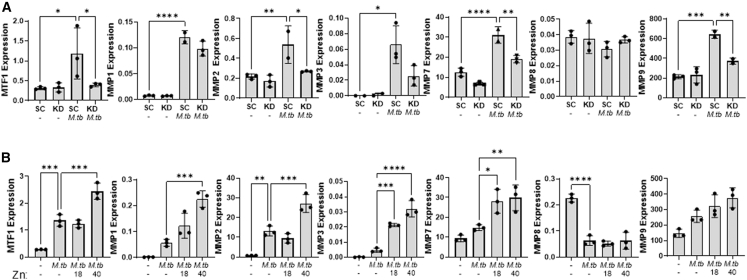
ZIP8 is important for MTF-1 and MMP expression during *M.tb* infection of macrophages (A) MDMs were transfected with scrambled control (sc) or ZIP8 (KD) siRNA, then infected with *M.tb* (MOI 5) for 48 h. (B) MDMs were infected with *M.tb* (MOI 5) for 2 h, washed, and incubated in the presence or absence of the indicated concentration of Zn (μM) for 48 h. (A and B) RNA was collected, and mRNA expression was assessed by qRT-PCR. Results are mean ± SD, representative of *n* = 3 independent biological replicates with 2–3 technical replicates each. One-way ANOVA with Dunnett’s post-test, ∗*p* < 0.05, ∗∗*p* < 0.01, ∗∗∗*p* < 0.001, and ∗∗∗∗*p* < 0.0001. Each dot indicates a technical replicate.

In addition to NF-κB driving inflammatory responses, MTF-1 is activated by Zn and is a key transcription factor driving MMP expression.^14^ Since MMPs are induced during *M.tb* infection and associated with disease severity,^37^^,^^38^ we next determined whether ZIP8 contributes to *M.tb*’s ability to drive MTF-1 and MMP expression in human macrophages. *M.tb* induced the expression of MTF-1, MMP1, MMP2, MMP3, MMP7, and MMP9 and this was reduced with ZIP8 KD (Figure 4A). Intriguingly, MMP8 was the only MMP not induced by *M.tb*, nor was it affected by ZIP8 KD (Figure 4A). Since MTF-1 and MMP activities are specific to Zn and not to other divalent ions,^39^ we posit that this is due to ZIP8-mediated Zn transport driving MTF-1 expression and activity, leading to MMP expression. Indeed, the addition of Zn led to increased MMP1,2,3,7, and 9 expression (Figure 4B). Thus, our data indicate that ZIP8 has disparate impacts on cytokine and MMP expression, dampening protective IL-6, IL-1β, TNFα, and IL-8 expression while conversely driving MMP expression, both of which would favor bacterial growth in macrophages.

### Zn dampens reactive oxygen species (ROS) in macrophages

In addition to MMPs, MTF-1 drives the expression of metallothioneins, which scavenge reactive oxygen species (ROS)^5^ and dampen host immune responses in murine macrophages.^40^ We therefore next determined whether Zn dampens the ROS burst in human macrophages. We found that Zn addition significantly reduced both basal and PMA-induced ROS burst in primary human macrophages after 10 min post Zn or PMA addition (Figure S4A). This was maintained through at least 30 min and observed with different PMA concentrations (Figure S4B). Most importantly, this was also observed during *M.tb* infection, *M.tb* induced a relatively low level of ROS (Figure S5A), which Zn reduced (Figure S5B). These results indicate that Zn dampens the ROS burst in human macrophages during *M.tb* infection, which, along with dampening pro-inflammatory cytokines, is expected to enhance *M.tb* intracellular survival.

### ZIP8 is important for *M.tb* growth in macrophages

**Figure 5:**
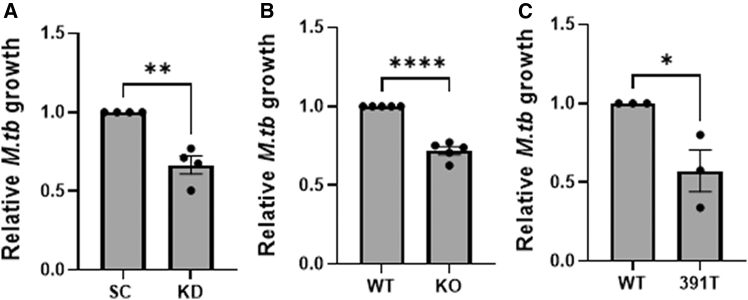
ZIP8 is important for *M.tb* growth in macrophages (A) MDMs were transfected with scrambled control (sc) or ZIP8-specific (KD) siRNA, then infected with *M.tb* (MOI 5). After 48 h, cells were lysed and CFU enumerated. B) Bone marrow-derived macrophages (BMDMs) were isolated from WT and conditional *Zip8* KO mice. Macrophages were infected with *M.tb*-lux at MOI 5. After 48 h, relative luminescence units (RLU) were assessed. (C) BMDMs were isolated from WT or A391T hypomorphic variant mice. Macrophages were infected with *M.tb* (MOI 5). After 48 h, cells were lysed and CFU enumerated. (A–C) Results are mean ± SEM of *n* = 3 (C), 4 (A), or 5 (B) independent biological replicates with 3 technical replicates each, unpaired *t* test, ∗*p* < 0.05, ∗∗*p* < 0.01, and ∗∗∗∗*p* < 0.0001. Each dot indicates an independent biological replicate.

Altogether, ZIP8-mediated reduction of phagosomal Zn levels, protective pro-inflammatory cytokines, and ROS production is expected to enable *M.tb* growth in macrophages. Indeed, *M.tb* growth was significantly reduced in human macrophages following ZIP8 KD, by 33 ± 6% after just 48 h (Figures 5A and S6A). We also observed a small reduction in *M.tb* growth in murine macrophages deficient in ZIP8 expression (Figure S6B). Since this reduction in CFUs was not striking, we repeated these experiments with a more sensitive luminescent *M.tb* reporter strain to provide another readout for *M.tb* growth and observed a more marked reduction in *M.tb* luminescence with ZIP8 KO, with a statistically significant reduction of 28 ± 3% after 48 h (Figures 5B and S6C). To complement these studies, we obtained macrophages from a mouse model engineered to express the common hypomorphic human ZIP8 A391T variant.^16^ Consistent with the ZIP8 KD and KO studies, *M.tb* growth was significantly reduced in murine macrophages obtained from homozygous hypomorphic defective carriers of the ZIP8 variant, by 43 ± 13% after 48 h (Figures 5C and S6D). We note that reduced bacterial growth with ZIP8 KD/KO was not due to cell death and monolayer loss, since ZIP8 KD led to increased cytokine expression and release (Figure 3 and S2), and similar macrophage numbers were observed between groups even after 72 h of infection (Figure S6E). These data provide evidence that ZIP8 contributes to *M.tb* growth in human and mouse macrophages.

### ZIP8 regulates the immune response during *M.tb* infection in mice

**Figure 6:**
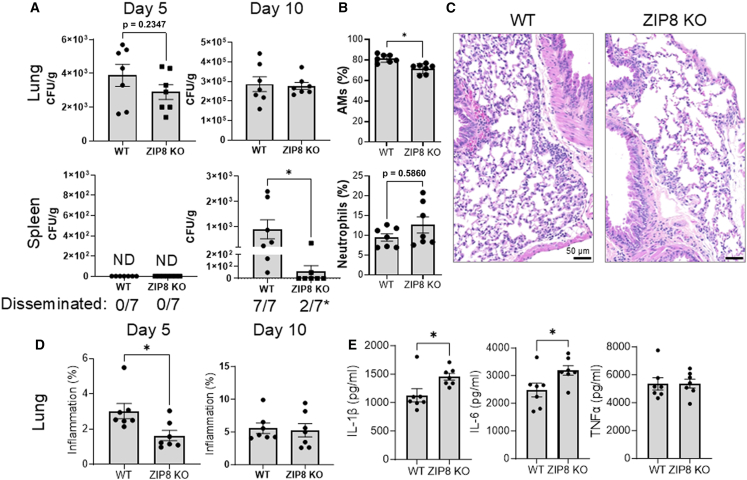
ZIP8 contributes to *M.tb* dissemination and the immune response *in vivo* (A–E) Mice were aerosol infected with 100 *M.tb*. (A) CFU in lungs and spleen were enumerated after 5 or 10 days, ND = not detected. (B) Flow cytometry analysis of lung digests was performed to determine the relative percentage of alveolar macrophages (AMs, CD45^+^CD11c+SiglecF+) and neutrophils (CD45+Ly6G+) in the lungs after 5 days of *M.tb* infection. (C and D) 2–3 non-consecutive lung slices were H and E stained (C, scale bars, 50 μm, 5 days after infection), and HALO software was used to quantify the mean % inflamed lung for each mouse (D). (E) Cytokine levels in the lungs were assessed after 5 days. (A–E) Each dot indicates one mouse. Results are mean ± SEM, representative of at least two independent experiments, with 7 mice/group/experiment, unpaired *t* test, ∗*p* < 0.05.

To determine the impact of ZIP8 on macrophage function *in vivo*, we infected myeloid-specific *Zip8* KO^28^^,^^29^ and control mice with *M.tb* by aerosolization and focused on the early host response to infection within the first 10 days, before adaptive immunity is initiated. *Zip8* KO led to a moderate reduction in *M.tb* burden in the lungs after just 5 days of infection (Figure 6A). We were unable to detect *M.tb* in the spleen after 5 days, but observed significantly less dissemination to the spleen after 10 days in the *Zip8*KO mice (Figure 6A). We have previously reported that the lungs of uninfected adult *Zip8*KO mice are identical to WT counterparts based on BAL cell counts, tissue architecture, and histology, with no evidence of inflammation.^28^^,^^29^ In contrast, during *M.tb* infection, *Zip8*KO mice had a small, but significant, reduction in the number of alveolar macrophages in the lungs (Figure 6B), and the extent of tissue inflammation was low. We also observed significantly less mononuclear and histiocytic inflammation in the alveolar interstitium in infected *Zip8*KO mice (Figures 6C and 6D). The reduction in *M.tb* burden and inflammation did not correspond with reduced inflammatory cytokines. After 5 days of infection, *Zip8*KO mice produced significantly more IL-1β and IL-6, while TNFα levels were unchanged (Figure 6E), consistent with our *in vitro* data that ZIP8 KD macrophages have increased NF-κB activity and inflammatory cytokine expression and release (Figures 3 and S2). These results indicate that although the levels of alveolar macrophages and inflammation are reduced in the lungs early during *M.tb* infection, the reduced immune cells that are present have increased NF-κB activity and increased expression and release of IL-1β and IL-6 in *Zip8*KO mice at this early time point. These data provide further support for a role of ZIP8 in regulating host immune responses and *M.tb* growth *in vitro* in macrophages and *in vivo* in mice.

## Discussion

**Figure 7:**
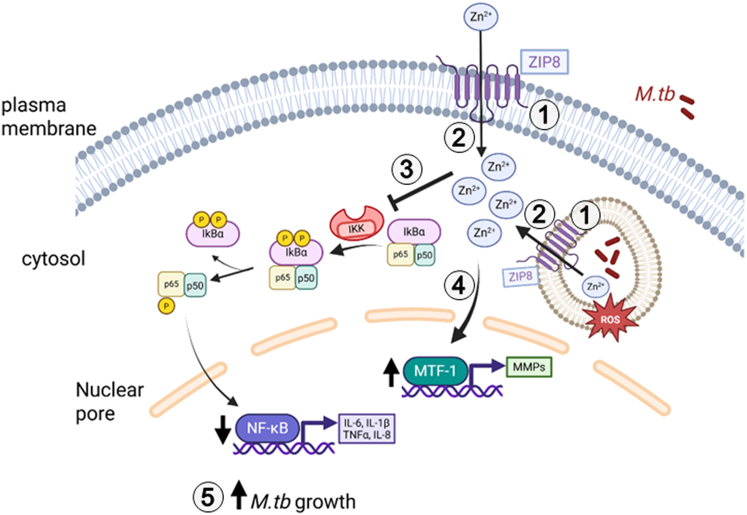
Model During *M.tb* infection of primary human macrophages, ZIP8: (1) localizes to the plasma membrane and *M.tb* phagosomal membrane within just 10 min, (2) imports Zn into the macrophage cytosol and out of the *M.tb* phagosome, (3) dampens NF-κB activity and pro-inflammatory cytokine expression, (4) increases MTF-1 and MMP expression, and ultimately (5) allows for enhanced *M.tb* intracellular growth and dissemination. Figure created with BioRender. Matouba, D. (2026) https://BioRender.com/gd3v45p.

Zn is essential for healthy immunity and prokaryotic and eukaryotic cell survival, thereby creating a “tug of war” for nutrient acquisition between host cells, including macrophages, and pathogens such as *M.tb*. Here, we show that in primary human macrophages the Zn importer ZIP8: 1) rapidly co-localizes with *M.tb* in the phagosome (within just 10 min), 2) exports Zn out of the *M.tb* phagosome and into the macrophage cytosol, 3) dampens NF-κB activity and pro-inflammatory cytokine expression, 4) increases MTF-1 and MMP expression, and ultimately 5) allows for enhanced *M.tb* intracellular growth and dissemination. Cytosolic Zn dampens the expression of pro-inflammatory cytokines and ROS and increases MTF-1 and MMP expression during *M.tb* infection (Figure 7).

We have previously shown that Zn, through ZIP8, decreases LPS-induced inflammation and pro-inflammatory cytokine production (IL-6, IL-1β, TNFα, IL-8) in LPS-stimulated monocytes by inhibiting IKKβ and NF-κB activity.^36^ Recently, others have also reported that increased intracellular Zn during Salmonella infection of murine macrophages dampens NF-κB activity and cytokine expression.^41^ Altogether, these findings highlight the important role of Zn transporters and Zn in regulating the human and murine macrophage response to infection/stimulation. Consistent with our previous work, we note that ZIP8 KD only moderately increased p65 and IκBα phosphorylation. This could be due to the Zn upregulation of A20 expression and/or phosphodiesterase (PDE) inhibition, which can also inhibit NF-κB.^3^

*M.tb* drives the expression of MMPs, which are linked to increased tissue damage in patients with TB.^37^^,^^38^ Here, we show that ZIP8-mediated transport of Zn contributes to *M.tb*-induced MTF-1 and MMP expression in human macrophages. Previous work identified the importance of the Zn-ZIP8-MTF1 axis in chondrocytes during osteoarthritis,^42^ and here we show that this occurs in macrophages in response to an infection. MTF-1, MMP1, MMP2, MMP3, MMP7, and MMP9 were all induced by *M.tb*. In sharp contrast, MMP8 was not induced by *M.tb*, and was the only MMP unchanged with ZIP8 KD or Zn stimulation, suggesting that the expression of this MMP is regulated differently than others. Indeed, although increased MMP8 is observed in patients with TB, neutrophils, and not macrophages, are the main producers of MMP8 during infection.^38^ Both MTF-1 and MMPs require Zn for activity. MTF-1 is a Zn finger protein that recognizes metal-response elements in promoters of metal-responsive genes such as the MMPs,^14^ and Zn is a cofactor of MMPs.^39^ Thus, Zn may drive MTF-1 and MMP expression and function both transcriptionally and post-translationally, in a positive feedback loop. MTF-1 also drives the expression of metallothioneins, which can scavenge ROS,^5^ providing a potential mechanism for how Zn dampens ROS during *M.tb* infection. In addition, Zn activates antioxidants glutathione and superoxide dismutase (SOD) and inhibits NADPH oxidase activity,^5^ which likely contributed to the rapid Zn-mediated dampened ROS burst observed here.

*M.tb* has adapted to respond to both low and high Zn levels to enable survival. *M.tb* produces kupyaphores, which bind Zn^43^ and Rip1, an intramembrane protease, both of which are important for growth in low Zn environments.^44^ *M.tb* also expresses transporters to allow for growth in high Zn environments.^11^^,^^45^ We show that Zn levels rapidly increase in the *M.tb* phagosome, and this is maintained through 68 h. This is supported by other reports showing increased phagosomal Zn during the first 24 h of infection.^11^^,^^12^ However, the host factors involved in regulating phagosomal Zn levels were unknown. We have shown that TNF, LPS, BCG, and *M.tb* increase ZIP8 expression.^4^^,^^18^ Here, we show that ZIP8 rapidly co-localizes to the *M.tb* and BCG phagosome, and this is maintained for at least 48 h. Further, we show that the *M.tb* expression of the Zn-regulated gene CtpC increases in response to ZIP8 KD, indicating that ZIP8 KD further increases Zn levels in the phagosome. Thus, both *M.tb* CtpC and host ZIP8 may be induced and localized by *M.tb* to moderate bacterial and phagosomal Zn levels, respectively, and allow for improved *M.tb* growth, averting the host's attempt to directly kill the bacteria through Zn poisoning. Zn dampens *M.tb* EsxG-EsxH expression and release, which inhibit phagosome lysosome fusion and are required for *M.tb* survival in macrophages and mice.^46^ It is interesting to speculate that increased phagosomal Zn would reduce *M.tb* EsxG-EsxH expression and thereby increase phagosome lysosome fusion. Altogether, ZIP8 is induced by *M.tb,* perhaps as part of the innate immune response, which inadvertently reduces phagosomal Zn that facilitates improved growth. We have previously shown that with extracellular bacteria, the induction of ZIP8 in macrophages is essential to infection resolution.^28^ In contrast, in the setting of *M.tb* infection, targeted inhibition of ZIP8 expression that leads to increased phagosome Zn levels may foster innovative approaches to control *M.tb* growth. In support of this, a recent study that screened compounds that effectively reduced *M.tb* growth in human macrophages identified a compound that works by increasing phagosome Zn levels in macrophages.^34^

Altogether, we show that ZIP8 is a susceptibility factor for *M.tb*, and identify several key pathways regulated by ZIP8 that contribute to ZIP8-mediated increased *M.tb* growth. This has important implications for individuals with dysfunctional ZIP8, including those with hypomorphic ZIP8, which may have increased resistance to *M.tb* infection. These data support the concept that ZIP8 inhibition may represent a promising target for host-directed therapy for TB and that future research carefully assessing ZIP8 inhibitors (including potential off-target effects, toxicity, or consequences for host tissue homeostasis) is warranted. Indeed, studies are underway to develop inhibitors for Zn transporters. A selective ZIP14 inhibitor that does not inhibit ZIP8 has recently been described, which improves mouse survival in a model of cancer cachexia, indicating that inhibitors specific to one ZIP are feasible and can be developed and safely used *in vivo*, although ZIP8 inhibitors are still in their infancy.^47^^,^^48^^,^^49^^,^^50^

### Limitations of the study

Here, we focused on the role of ZIP8 in macrophages since they are the primary niche for *M.tb* and assessed cytokine, MMP, and ROS pathways. Future studies are required to interrogate the impact of ZIP8 and Zn on other macrophage responses and in other cell types, as well as *M.tb* factors during *M.tb* infection. We report that ZIP8 co-localizes with intracellular *M.tb* and attenuated BCG. It remains unknown if ZIP8 also co-localizes with other intracellular pathogens. We focused specifically on ZIP8 since it is the only transporter that is induced during *M.tb* infection of human macrophages and implicated in immune functions. However, it is possible that other Zn transporters also regulate macrophage responses to *M.tb* infection. In addition to Zn, ZIP8 can transport manganese and iron. Although we confirmed that Zn regulates cytokine and MMP levels, we cannot rule out the possibility that ZIP8-mediated transport of other metals may also be involved in macrophages and their interactions with *M.tb*. Finally, we did not assess the impact of sex or age on ZIP8 and Zn function in macrophages. These are all important questions to assess in the future.

## Resource availability

### Lead contact

Requests for further information and resources should be directed to and will be fulfilled by the lead contact, Larry S Schlesinger (lschlesinger@txbiomed.org).

### Materials availability

This study did not generate new unique reagents.

### Data and code availability

All data produced in this study are included in the published article and its supplementary information and will be shared by the lead contact upon request. This paper does not report original code. Any additional information required to reanalyze the data reported in this paper is available from the lead contact upon request.

## Acknowledgments

The authors would like to acknowledge Jordan Bonifacio for her assistance and the facilities and programmatic support of the Biocontainment Program and Biology Core at Texas Biomed. This work was supported by the 10.13039/100000002National Institutes of Health [AI136831, 1P30AI168439, S10OD028732, AI132422, R03DK134595], and a Texas Biomedical Research Institute Forum Award. The funding sources were not involved in study design; collection, analysis, or interpretation of data; writing; or the decision to submit the manuscript. Graphical abstract created in BioRender. Matouba, D. (2026) https://BioRender.com/ggnppya.

## Author contributions

Conceptualization, E.A., C.P., D.K., and L.S.S.; formal analysis, E.A., M.L., E.H., D.C., C.M.L.W., S.P., D.M., B.D., C.R., B.L., O.G., V.S., E.J.D., A.A., and D.S.; methodology, E.A. and D.K.; investigation, E.A., M.L., E.H., D.C., C.M.L.W., S.P., D.M., B.D., C.R., B.L., A.A., C.P., D.S., and E.G.; visualization, E.A. and D.M.; writing – original draft, E.A.; writing – review and editing, E.A., M.L., C.M.L.W., S.P., D.M., B.L., O.G., V.S., E.J.D., A.A., A.K.O., J.M., D.K., and L.S.S.; funding acquisition, E.A., E.D.J., and L.S.S.; resources, V.T., A.K.O., J.M., and D.K.; supervision, E.A., D.K., and L.S.S.

## Declaration of interests

The authors declare no competing interests.

## STAR★Methods

### Key resources table

**Table:** 

REAGENT or RESOURCE	SOURCE	IDENTIFIER
**Antibodies**		

PerCP/Cyanine5.5 anti-mouse CD45 Antibody	BioLegend	Cat# 157208, Clone S18009F; RRID: AB_2860728
PerCP/Cyanine5.5 Rat IgG2b, κ Isotype Ctrl Antibody	BioLegend	Cat# 400632, Clone RTK4530; RRID: AB_893677
Alexa Fluor® 700 anti-mouse Ly-6G Antibody	BioLegend	Cat#127622, Clone 1A8; RRID: AB_10643269
Alexa Fluor® 700 Rat IgG2a, κ Isotype Ctrl	BioLegend	Cat#400528, Clone RTK2758; RRID: AB_2923249
APC anti-mouse CD11c Antibody	BioLegend	Cat# 117310, Clone N418; RRID: AB_313779
APC Armenian Hamster IgG Isotype Ctrl Antibody	BioLegend	Cat# 400912, Clone HTK888; RRID: AB_2905474
PE/Cyanine7 anti-mouse CD170 (Siglec-F) Antibody	BioLegend	Cat# 155528, Clone S17007L; RRID: AB_2890715
PE/Cyanine7 Rat IgG2a, κ Isotype Ctrl Antibody	BioLegend	Cat# 400522, Clone RTK2758; RRID: AB_326542
TruStain FcX™ PLUS (anti-mouse CD16/32) Antibody	Biolegend	Cat# 156604; RRID: AB_2783138
Rabbit anti-ZIP8	ProteinTech	Cat# 20459-1-AP; RRID: AB_10697830
Rabbit isotype	Abcam	Cat# ab172730; RRID: AB_2687931
Rabbit anti-phospho-p65	Cell Signaling	Cat# 3033, clone 93H1; RRID: AB_331284
Rabbit anti-p65	Cell Signaling	Cat# 4764, clone C22B4; RRID: AB_823578
Rabbit anti-phospho-IκBα	Cell Signaling	Cat# 2859, clone 14D4; RRID: AB_561111
Rabbit anti-IκBα	Cell Signaling	Cat# 4812, clone D44D4; RRID: AB_10694416
Rabbit anti-β-actin	Cell Signaling	Cat# 5125, clone 13E5; RRID: AB_1903890
Alexa Fluor® 488 chicken anti-rabbit	Invitrogen	Cat# A21441; RRID: AB_2535859
HRP goat anti-rabbit	Cell Signaling	Cat# 7074; RRID: AB_2099233

**Bacterial and virus strains**		

*M.tb* H_37_R_v_	ATCC	ATCC 27294
*M.tb* H_37_R_v_ lux	Salunke et al.^51^	N/A
mCherry *M.tb* H_37_R_v_	Dr. Sarah Fortune	N/A
*M.tb* Zn reporter strain	Li et al.^6^	N/A
*M.tb* Erdmann	Dr. Marcus Horwitz	N/A
mChery BCG	This manuscript	N/A

**Biological samples**		

MDMs	This manuscript	N/A
BMDMs	This manuscript	N/A

**Chemicals, peptides, and recombinant proteins**		

Ficoll-paque PLUS	GE healthcare	Cat# 17-1440-03
Amphotericin B	Sigma	Cat# A4888
Cycloheximide	Sigma	Cat# C7698
Trimethoprim	Sigma	Cat# T0667
Carbenicillin	Sigma	Cat# C1389
Vancomycin	Sigma	Cat# V2002
Polymyxin B	Sigma	Cat# P1004
4% Paraformaldehyde	Thermo Scientific	Cat# AAJ19943K2
Tris(2-pyridylmethyl)amine (TPA)	Sigma	Cat# 723134
Zn sulfate	Sigma	Cat# Z0251
FluoZin-3	Invitrogen	Cat# F24195
H2DCFDA (DCF)	Invitrogen	Cat# D399
Phorbol 12-myristate 13-acetate (PMA)	MP Biomedicals	Cat# MP218388201

**Critical commercial assays**		

Mouse Il-1β ELISA	R&D Systems	Cat# DY401
Mouse IL-6 ELISA	R&D Systems	Cat# DY406
Mouse TNFα ELISA	R&D Systems	Cat# DY410
Human Il-1β ELISA	R&D Systems	Cat# DY201
Human IL-6 ELISA	R&D Systems	Cat# DY206
Human TNFα ELISA	R&D Systems	Cat# DY210
Luminex assay	R&D Systems	Mouse Luminex Discovery Assay LXSAMSM-09 and -04 plex
Pierce BCA assay	Thermo Scientific	Cat# 23225
Direct-zol RNA Microprep Kit	Zymo Research	Cat# R2062

**Experimental models: Cell lines**		

NCTC clone 929, L-929 cells	ATCC	ATCC CCL-1

**Experimental models: Organisms/strains**		

Mouse: Conditional Zip8 KO (under LysM promoter)	Knoell et al.^28^^,^^29^	N/A
Mouse: C57BL/6J wild-type	Knoell et al.^28^^,^^29^ Sunuwar et al.^16^	N/A
Mouse: Hypomorphic ZIP8 A391T	Sunuwar et al.^16^	N/A

**Oligonucleotides**		

ZIP8 siRNA: TAGGAGTTAGGAAATAAATAA	Qiagen	Cat# 1027418, GeneGlobe ID: SI00724675
scrambled control siRNA: AATTCTCCGAACGTGTCACGT	Qiagen	Cat# 1027418, GeneGlobe ID: SI03650318
*M.tb* CtpC primer F: AGC AAG TGC TGG CCT ATG R: TGT GGT GGG ATG CTG ATG	IDT	N/A
*M.tb* 16S primer F: CCG GAA TTA CTG GGC GTA AA R: AGT ACT CTA GTC TGC CCG TAT C	IDT	N/A
Il1b primer	Applied Biosystems	Cat# Hs00174097_m1
Il6 primer	Applied Biosystems	Cat# Hs00174131_m1
Cxcl8 primer	Applied Biosystems	Cat# Hs00174103_m1
Tnf primer	Applied Biosystems	Cat# Hs00174128_m1
Mtf1 primer	Applied Biosystems	Cat# Hs00232306_m1
Mmp1 primer	Applied Biosystems	Cat# Hs00899658_m1
Mmp2 primer	Applied Biosystems	Cat# Hs01548727_m1
Mmp3 primer	Applied Biosystems	Cat# Hs00968305_m1
Mmp7 primer	Applied Biosystems	Cat# Hs01042796_m1
Mmp8 primer	Applied Biosystems	Cat# Hs01029057_m1
Mmp9 primer	Applied Biosystems	Cat# Hs00957562_m1
Actb primer	Applied Biosystems	Cat# Hs01060665_g1

**Software and algorithms**		

Belysa™ Immunoassay Curve Fitting Software	Millipore Sigma	N/A
Halo software	Indica labs	N/A
FlowJo 10.8.1 software	BD Biosciences	N/A
FIJI software	Open source	https://fiji.sc/
Gen5 software	BioTek	N/A
VisionWorks software	Analytik Jena	N/A
Prism software	GraphPad	N/A

**Other**		

Teflon JAR 4 fL. Oz./120 mL (holds 13–15 mL volume)	Savillex	Cat# 100-0120-01
Teflon well lids, 70 mm closure	Savillex	Cat# 600-070-01
Cell Staining Buffer	Biolegend	Cat# 420201
BD Horizon Brilliant Stain Buffer Plus	BD Biosciences	Cat# 566385
TransIT-X2 Transfection reagent	Mirus Bio	Cat# MIR6000
DAPI	Invitrogen	Cat# D1306
ProLong Gold Antifade	Invitrogen	Cat# P36930
Clarity ECL Reagent	BioRad	Cat# 170-5060
TRIzol	Invitrogen	Cat# 15596018
SuperScript III Reverse Transcriptase	Invitrogen	Cat# 18080085
PowerUp™ SYBR™ Green Master Mix	Applied Biosystems	Cat# A25742

### Experimental model and study participant details

#### *Zip8* KO and A391T mice

All mouse studies followed Texas Biomed approved Institutional Animal Care and Use Committee protocols 1703MU and 1695MU. Mice were group housed and kept under controlled conditions in a specific pathogen-free environment, with sterile food and water *ad libitum*. Conditional *Zip8* KO (under LysM promoter) mice were generated as previously described and bred in house.^28^^,^^29^ C57BL/6J wild-type (WT) counterparts were purchased from The Jackson Laboratory and bred in house for experimental procedures. All mice were genotyped, then a mix of female and male mice, 7–18 weeks old, were randomly assigned to experimental groups. Hypomorphic ZIP8 A391T variant mice were generated as previously described and C57BL/6J WT used as controls.^16^ BMDMs were obtained from tibias and femurs of 7–16 week-old C57BL/6J control and conditional ZIP8 knockout mice,^28^^,^^29^ and from a mouse model of human ZIP8 A391T (murine ZIP8 393T knock-in mice equivalent to human ZIP8 391T^16^) as previously described.^52^ BMDMs were frozen in DMEM supplemented with 30% L-cell conditioned media, 20% heat inactivated-fetal bovine serum (HI-FBS), 10 units penicillin/streptomycin (Pen/Strep), 50.3 μM β-mercaptoethanol and 10% dimethyl sulfoxide. L-cell conditioned media was obtained by collecting media from confluent NCTC clone 929 (ATCC # CCL-1) cells. The day before the experiment, BMDMs were thawed and plated in tissue culture dishes in DMEM supplemented with 10 units Pen/Strep and 10% HI-FBS. The day of the experiment, cells were washed twice with DMEM to remove the antibiotics from the culture medium.

#### Isolation and culture of human MDMs

Peripheral blood mononuclear cells (PBMCs) were isolated from human peripheral blood collected from male and female 18-50-year-old healthy donors, following Texas Biomed approved IRB protocol HSC20170315H. All donors for these studies provided informed, written consent. MDMs were prepared as described elsewhere.^53^^,^^54^ Briefly, heparinized blood was layered on a Ficoll-Paque cushion (GE Healthcare, Uppsala, Sweden) to allow for collection of PBMCs. PBMCs were cultured in RPMI (Life Technologies, Carlsbad, CA) with 20% autologous serum in Teflon wells (Savillex, Eden Prairie, MN) for 5–6 days at 37°C/5% CO_2_. MDMs were harvested and adhered to tissue culture dishes for 2–3 h in RPMI with 10% autologous serum, lymphocytes were washed away, and MDMs were incubated overnight in RPMI with 10% autologous serum. Such MDM monolayers are 99% pure and viable. A minimum of three different experiments with independent biological replicates was performed, with macrophages from at least three different human donors, unless indicated otherwise in the figure legend.

#### Bacterial strains

Lyophilized *M.tb* H_37_R_v_ (27294) were obtained from the American Tissue Culture Collection (ATCC, Manassas, VA). *M.tb* H_37_R_v_ lux was created and used as described.^51^ This bacterial strain contains the entire bacterial Lux operon cloned in a mycobacterial integrative expression vector, and luciferase activity is correlated with bacterial growth.^51^^,^^55^^,^^56^ mCherry *M.tb* H_37_R_v_ was a kind gift from Dr. Sarah Fortune (Harvard University, Boston, MA). mCherry BCG was generated by introducing a mCherry-expressing plasmid (kindly provided by Dr. Fortune^57^) into *M. bovis* BCG. The *M.tb* Zn reporter strain was generated as described previously.^6^ For *in vitro* infections, bacteria were cultured on 7H11 at 37°C for 9–14 days, then single cell suspensions of bacteria were prepared as previously described.^58^^,^^59^ The bacteria concentration and degree of clumping (<10%) were determined with a Petroff-Hausser Chamber. This method results in ≥90% viable bacteria, as determined by CFU assay. Killed *M.tb* was prepared by incubating with 4% paraformaldehyde (PFA) for 20 min at room temperature and then washed with PBS.^33^

### Method details

#### *M.tb* infection of macrophages

Single cell suspensions of *M.tb* in RHH [for MDM infections: 10 mM HEPES (Life Technologies) and 0.1% human serum albumin (CSL Behring, King of Prussia, PA) in RPMI] or DHH [for BMDM infections: 10 mM HEPES (Life Technologies) and 0.1% human serum albumin (CSL Behring) in DMEM] were added to the macrophages at various MOIs and cells were incubated for 2 h at 37°C, with the first 30 min on a platform shaker. Macrophages were then washed and incubated in RPMI with 2% autologous serum (for MDMs) or DMEM with 2% HI-FBS (for BMDMs) for the indicated times. For synchronized phagocytosis, after *M.tb* were added to MDMs the cells were centrifuged at 350g for 5min at 4°C, then incubated at 37°C/5% CO_2_.

#### *M.tb* infection of mice

*M.tb* Erdmann was grown in Proskauer Beck medium containing 0.01% Tween 80 to mid-log phase and stored at −80°C.^60^ The number of bacteria were determined by plating for CFUs. Mice were aerosol infected with 100 *M.tb* CFU using an Inhalation Exposure System (Glas-col, Terre Haute, IN).^60^ After 5 or 10 days of infection mice were euthanized using CO_2_ asphyxiation. Spleens and lungs were collected and homogenized to assess *M.tb* burden by plating serial dilutions onto OADC-supplemented 7H11 agar plates containing specific antibiotics/antifungals: Amphotericin B (20 mg/L), Cycloheximide (20 mg/L), Trimethoprim (20 mg/L), Carbenicillin (50 mg/L), Vancomycin (10 mg/L), and Polymyxin B (25 mg/L). *M.tb* CFUs were counted after 6 weeks at 37°C. Cytokine concentrations in cell free homogenates from the lung were assessed by ELISA (R&D Systems, Minneapolis, MN) following the manufacturer instructions or Luminex assay in the Luminex 100/200 System (Mouse Luminex Discovery Assay LXSAMSM-09 and -04 plex, Luminex Corporation) following the manufacturer’s protocol. The data were analyzed using Belysa Immunoassay Curve Fitting Software (Millipore Sigma). To assess inflammation, the left lung lobes from individual mice were inflated with 10% neutral buffered formalin, embedded in paraffin, then sliced to generate 2–3 non-consecutive slices, and stained with hematoxylin and eosin. Slides were digitally scanned using Zeiss Axio scan.Z1 (Zeiss, Germany). Halo software (V3.3, Indica labs, NM, USA) was used to quantify the portion of inflamed lung.

For flow cytometry analysis, the right lung lobe was dissociated into single cell suspensions by enzymatic degradation with collagenase Type I-A and type IV bovine pancreatic DNase 1 for 15 min at 37°C, 5% CO_2_ and 95% relative humidity followed by mechanical dissociation (gentleMACS dissociator, MiltenyiBiotec inc.). RPMI with 10% FBS was added to dilute enzymatic activity. Residual red blood cells were lysed using 1 mL of lysis buffer (0.15M NH_4_Cl, 1 mM KHCO_3_) for 2 min at room temperature followed by washing with RPMI. The lung cells were passed through sterile 70 μm nylon mesh screens (BD Biosciences; San Jose, CA) to obtain single cell suspensions. Cells were centrifuged at 300g for 10 min at 4°C and re-suspended in RPMI then placed into round-bottom polypropylene tubes (1-2 × 10^5^) and centrifuged at 250g for 10 min. The cell pellets were resuspended in 50 μL of cell staining buffer (Biolegend) along with TruStain FcX PLUS (anti-mouse CD16/32) Antibody from Biolegend for FC receptor blocking. After a 30 min incubation on ice, the cells were stained with fluorochrome-tagged antibodies and corresponding isotype-matched control antibodies in BD Horizon Brilliant Stain Buffer Plus for 40 min in the dark at 4°C. Following this, the cells were washed once, fixed in 4% PFA in cell staining buffer for 30 min, centrifuged (250g for 10 min), and then resuspended in 300 μL cells staining buffer. The cells were then filtered through a round-bottom polystyrene tube with a cell strainer snap cap (Falcon). The samples were acquired using a BD FACSymphony multi-color flow cytometer, and compensation, analysis, and data visualization were carried out using FlowJo 10.8.1 software (BD Biosciences). Isotype and “Fluorescence minus one” controls were utilized as needed to establish gates. The fluorochrome-tagged antibodies were purchased from BD Biosciences, San Jose, CA, and Biolegend, San Diego, CA.

#### Gene knockdown

MDMs were transfected with 50 nM ZIP8 siRNA (TAGGAGTTAGGAAATAAATAA) or scrambled control siRNA (AATTCTCCGAACGTGTCACGT) with TransIT-X2 Transfection reagent (Mirus, Madison, WI), following the manufacturer’s recommendations. All siRNAs were purchased from Qiagen. MDMs were incubated 48 h before use.

#### Fluorescence microscopy

##### ZIP8 and *M.tb*

Cells were fixed with 4% PFA, permeabilized with cold methanol, then labeled with ZIP8 specific antibodies (ProteinTech 20459-1-AP) and DAPI (Invitrogen). Coverslips were mounted with ProLong Gold Antifade (Invitrogen) and imaged with a Zeiss LSM 800 confocal microscope. ZIP8 colocalization with *M.tb* was assessed manually by counting at least 50 *M.tb* per experiment, and in HALO v3.3 (Indica Labs, USA) using the object colocalization module. The software module quantifies the absolute area of bacteria, ZIP8 and areas of colocalization of bacteria and ZIP8.

##### Zn

MDMs were treated with 1 μM TPA for 30 min to chelate Zn, 18 μM Zn sulfate (Sigma Z0251) for 2 h, or infected with *M.tb* at MOI 10 for 2 h. MDMs were then washed, fixed with 4% PFA, stained with 5 μM FluoZin-3 (Fisher F24195) and DAPI (Invitrogen), and imaged within a couple hours. Mean fluorescent intensity (MFI) of FluoZin-3 in 1,000 macrophages was automatically enumerated with FIJI. Images were background corrected, and a threshold was applied such that only the sum of the fluorescence intensities of the pixels located within the MDMs (SFI) was measured. The results were expressed as the mean fluorescence intensity (MFI) per cell according to: (MFI per cell) = (SFI)/N MDM. To quantify Zn on a per cell basis, images were analyzed with Gen 5 (Agilent, Santa Clara, CA).

##### *M.tb* Zn reporter

*In broth culture*. The *M.tb* Zn reporter strain was cultured in Sauton’s Media with or without 34.8 mM Zn until OD 500. Bacteria were centrifuged and resuspended in Sauton’s Media with Zn (34.8 mM) or EDTA (0.5 mM) to chelate Zn. Bacteria were incubated overnight at 37°C then fixed and imaged with a Zeiss LSM 800 confocal microscope. *In macrophages*. MDMs were infected with the *M.tb* Zn reporter strain at MOI 10, then the cells were centrifuged at 350g for 5min at 4°C and incubated at 37°C/5% CO_2_. After 2 h, cells were washed then cultured in 2% autologous serum. Live cell imaging was performed using the Cytation 5 microscope paired with BioSpa (Agilent) to maintain cells at 37°C/5% CO_2_ for the duration of the movie. Images were acquired every 4 h with a 60× objective and Dendra2 and mCherry signals of >1,000 bacteria were quantified.

#### Western blotting

Cells were washed with PBS, then lysed with TN1 lysis buffer (125 mM NaCl, 50 mM Tris, 10 mM EDTA, 1% Triton X-100, 10 mM Na_4_PO_7_, 10 mM NaF with 10 mM Na_3_VO_4_, 10 μg/mL aprotinin, and 10 μg/mL leupeptin) at 4°C. Lysates were centrifuged (10,000g, 4°C, 10 min) to remove cell debris, then a Pierce BCA assay (Thermo Scientific, Waltham, MA) was performed to determine protein concentration. Equivalent amounts of denatured and reduced protein were separated by SDS-PAGE and analyzed by Western blot using antibodies against ZIP8 (ProteinTech 20459-1-AP), p-p65 (Cell Signaling 3033), p65 (Cell Signaling 4764), p-IκBα (Cell Signaling 2859S), IκBα (Cell Signaling 4812S), and β-actin (clone 13E5, cat# 5125 Cell Signaling). The membranes were developed using clarity ECL reagent on a UVP ChemStudio 815 system (AnalytikJena US LLC, Upland CA). Protein band intensities were determined with VisionWorks. For each sample, background values were subtracted and then values were normalized to the β-actin, p65, or IκBα loading control as indicated.

#### RNA isolation and gene expression by qRT-PCR

Macrophages in triplicate wells were lysed with TRIzol (Invitrogen) and total RNA was isolated according to the manufacturer’s recommendations. The NanoDrop One^C^ was used to determine quantity and quality of RNA. cDNA was reverse transcribed from RNA with SuperScript III Reverse Transcriptase (Invitrogen). Macrophage gene expression was determined by quantitative real-time RT-PCR (qRT-PCR) using TaqMan Gene Expression Assays (Applied Biosystems, Foster City, CA) with predesigned and validated primers in the Applied Biosystems 7500 Real-Time PCR System. Relative expression was calculated by the ΔΔ threshold cycle (ΔΔCT) method using β-actin as the housekeeping gene.^56^^,^^61^ To assess *M.tb* gene expression, macrophages were lysed with TRIzol and total RNA was isolated using Direct-zol RNA Microprep Kit (Zymo Research). RNA was measured and cDNA reverse transcribed as above. *M.tb* gene expression was determined by qRT-PCR using PowerUp SYBR Green Master Mix (Applied Biosystems) in the 7500 Real-Time PCR System. The 7500 thermal cycler was operated as follows: Stage 1 (50°C for 2 min), stage 2 (95°C for 10 min), stage 3 (95°C for 15 s, 52°C for 20 s, 60°C for 1 min). Dissociation steps were added in Stage 4. Data were collected during stage 3, specifically at step 3. Relative expression was calculated by the ΔΔCT method using 16S as the *M.tb* housekeeping gene. No *M.tb* genes were detected in uninfected controls.^35^ The forward and reverse primers were procured from IDT. The primer sequences are listed in the table.

#### ELISAs

Cytokine concentrations in cell free supernatants were measured by ELISA (R&D Systems, Minneapolis, MN) following the manufacturer instructions.

#### DCF assay for detecting ROS burst

On the day of each experiment, MDMs were cultured in DPBS-HHG (10 mM HEPES, 0.1% human serum albumin, 0.1% glucose in DPBS with Ca, Mg) + 30 μM DCF (Invitrogen) for 30 min at 37°C. Cells were then stimulated with PMA (0.4, 1 μg/mL), ZnSO_4_, *M.tb* by synchronized phagocytosis, and/or medium-only control. For conditions of synchronized phagocytosis, MDMs were cooled to 4°C for 10 min before addition of *M.tb*, and *M.tb* were centrifuged onto MDMs at 350 g for 10 min. Cells were then warmed to 37°C, and fluorescence of DCF, indicative of ROS production, was measured every 2 min for a total of 60 min at 37°C using a Glomax Explorer plate reader (Promega, Madison, WI; *M.tb* infection) or SpectraMax M2 (Molecular Devices, San Jose, CA; PMA stimulation). Test conditions were measured in triplicate wells.

#### *M.tb* growth assays

Intracellular growth was assayed with CFU assays. Infected macrophages were lysed as described previously.^55^^,^^62^ Lysates were diluted and plated on 7H11 agar (Remel) supplemented with OADC (Oleic acid, Albumin, Dextrose, Catalase) and glycerol. The number of CFUs was enumerated after growth for 3–4 weeks at 37°C. For luciferase growth assays, macrophages were infected with *M.tb*-lux, and bacterial bioluminescence was measured in relative luminescence units (RLUs) every 24 h with a GloMax Microplate Reader (Promega, Madison, WI).^51^

### Quantification and statistical analysis

A minimum of three different experiments was performed, with macrophages from at least three different human donors (three independent biological replicates), unless indicated otherwise in the figure legends. Although the trend was the same for each donor, the magnitude of change differed among donors. Consequently, results from each experiment were normalized to an internal control and an unpaired one-tailed Student’s *t* test (if comparing two groups) or ANOVA (when comparing more than 2 groups) were performed on the normalized data using Graphpad Prism, with *p* < 0.05 considered significant, ∗*p* < 0.05, ∗∗*p* < 0.01, ∗∗∗*p* < 0.001, ∗∗∗∗*p* < 0.0001, statistical details can be found in the figure legends.
