# Supplementary material for: Mycobacterium tuberculosis subverts macrophage-mediated defense through exploitation of the Zn transporter ZIP8
Source: iScience. 2026 Jun 9;29(6):116276. doi: 10.1016/j.isci.2026.116276 (PMC13273473; doi:10.1016/j.isci.2026.116276)
Supplement: Document S1. Figures S1–S7 [file mmc1.pdf]

iScience, Volume *29*

Supplemental information

Mycobacterium tuberculosis subverts macrophage-mediated defense through exploitation of the Zn transporter ZIP8

Eusondia Arnett, Miranda Lumbreras, Elizabeth Hernandez, Daniela Campos, Chrissy M. Leopold Wager, Susanta Pahari, Dahlia Matouba, Brenden F. Determann II, Charles Renshaw, Brandon Longstreet, Olga Gonzalez, Vinay Shivanna, Edward J. Dick Jr., Abul Azad, Charlie Pyle, Deandra Smith, Vartika Tomar, Evelyn Guirado, Anil K. Ojha, Joanna Melia, Daren Knoell, and Larry S. Schlesinger

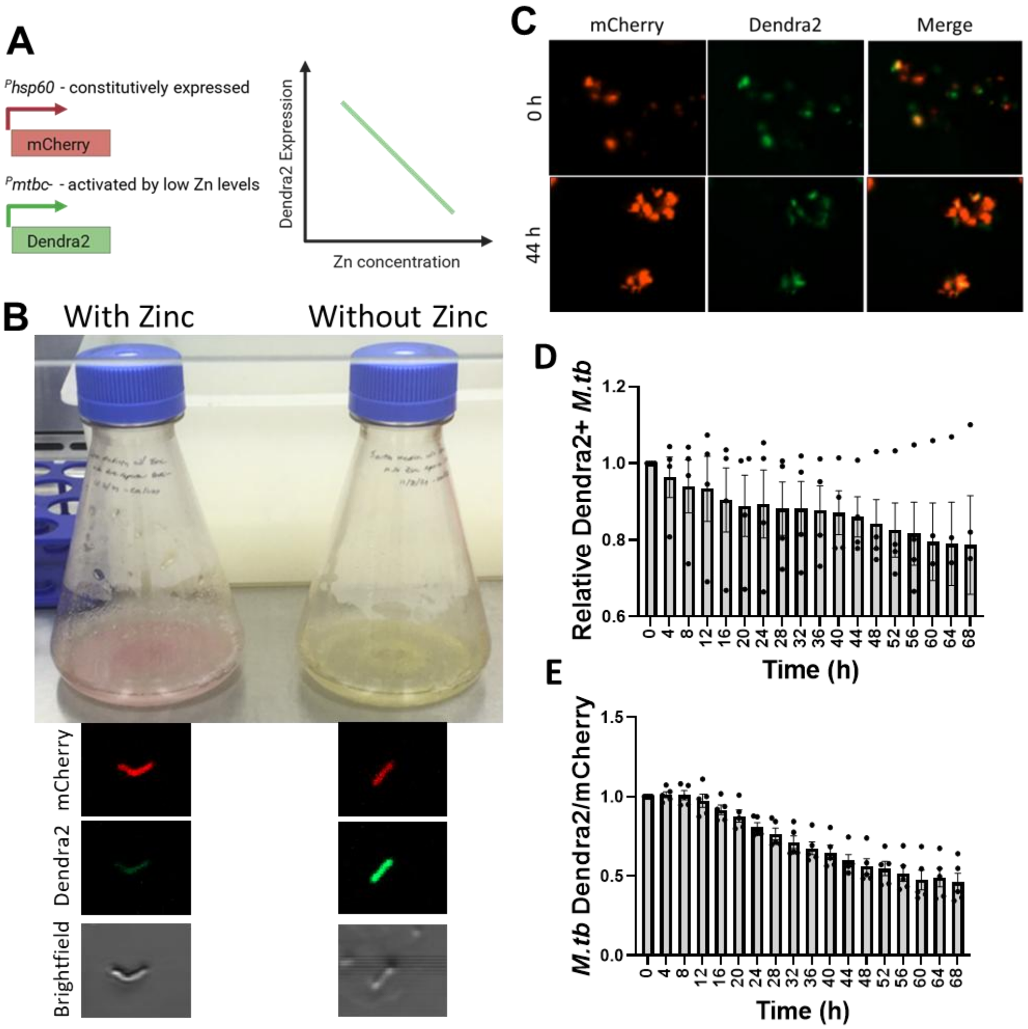

Fig S1. Zn levels increase in the *M.tb* macrophage phagosome. A) Schematic of the *M.tb* Zn reporter strain, with Dendra2 under control of the Zn-regulated *mtb*$_c$- promoter and mCherry under control of the constitutive hsp60 promoter. The illustration on the right shows the inverse correlation between Dendra2 expression and Zn concentration, with low Zn levels driving the *mtb*$_c$- promoter. Created in BioRender. Arnett, E. (2026) https://BioRender.com/y84w661. **B)** *M.tb* strains were cultured in Sauton's Media ± Zn (34.8 mM) until OD 500 and images acquired (top panel), then pelleted and cultured in Sauton's Media with Zn (34.8 mM) or EDTA (0.5mM) overnight before imaging wet mounts (bottom panel). **C-E)** MDMs were infected with the *M.tb* Zn reporter strain (MOI 10) for 2h, then washed and cultured in 2% autologous serum. Images were acquired every 4h. **C)** Representative images over time. **D)** Dendra2-positive *M.tb* were enumerated over time, at least 1,000 bacteria per experiment were quantified. Mean ± SEM of four independent biological replicates with two technical replicates each, each point indicates one biological replicate. **E)** Ratio of Dendra2 to mCherry MFI for at least 1,000 bacteria per experiment was quantified. Mean ± SEM of five independent biological replicates with two technical replicates each; each point is one biological replicate, relative to ratio at Time 0.

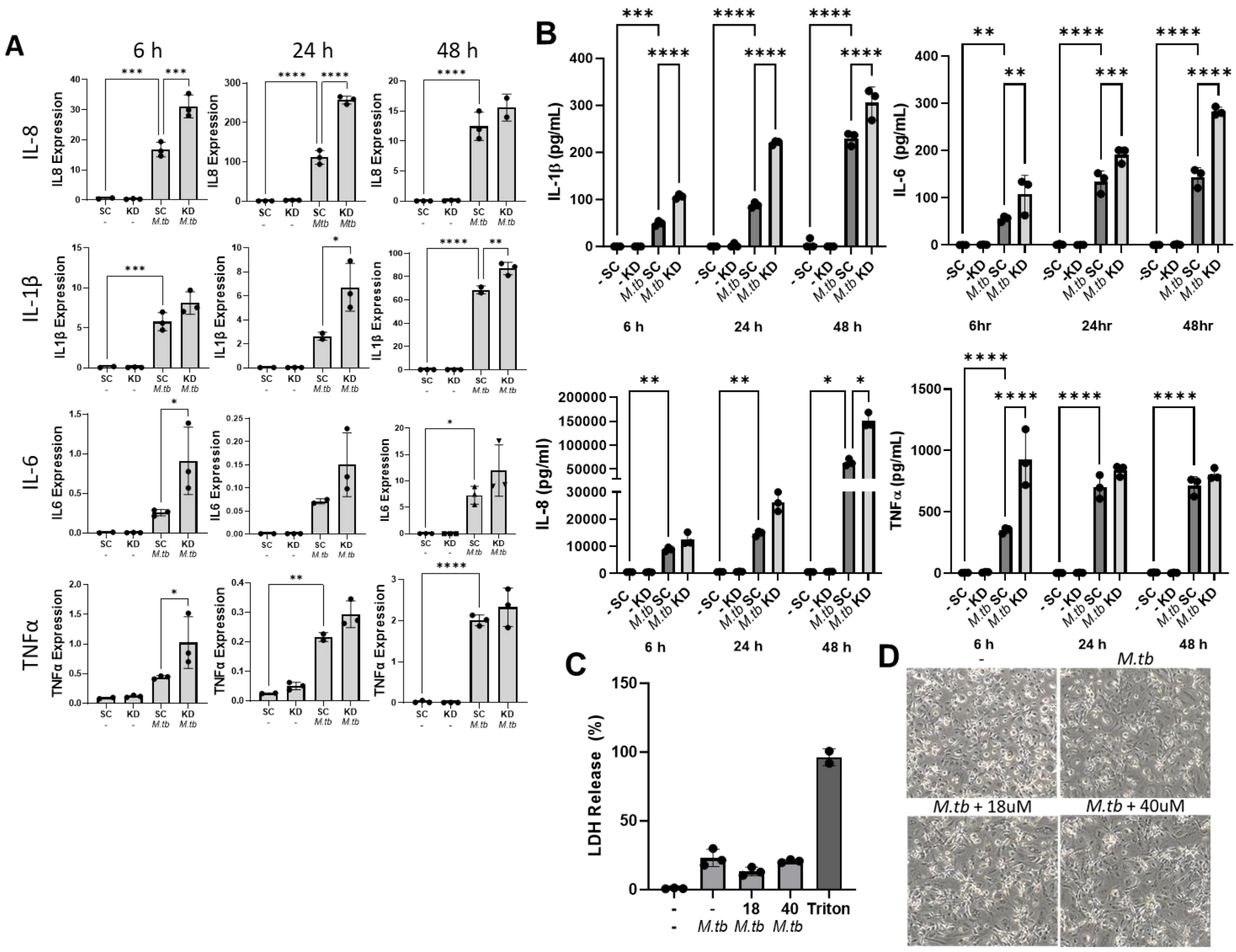

Fig S2. ZIP8 dampens pro-inflammatory cytokine release during *M.tb* infection of macrophages. A,B) MDMs transfected with scrambled control (sc) or ZIP8 (KD) siRNA were infected with *M.tb* (MOI 5) for 6-48h. **A)** RNA was collected, and mRNA expression was assessed by qRT-PCR. Results are mean ± SD of 2-3 technical replicates from n=3 (IL-1β, IL-6, TNFα: 24h; IL-8: 6, 24 and 48h), or 4 (IL-1β, IL-6, TNFα: 6 and 48h) independent biological replicates, each dot indicates results from one well. One-way ANOVA with Dunnett's post-test, * $p < 0.05$, ** $p < 0.01$, *** $p < 0.001$, **** $p < 0.0001$. **B)** Supernatant was collected, and cytokine release assessed by ELISA. Results are mean ± SD of 3 technical replicates from n=3 (24h) or 4 (6 and 48h) independent biological replicates, each dot indicates results from one well. Two-way ANOVA with Tukey's post-test, * $p < 0.05$, ** $p < 0.01$, *** $p < 0.001$, **** $p < 0.0001$. **C,D)** MDMs were infected with *M.tb* (MOI 5) for 2h, washed, and incubated in the presence or absence of the indicated concentration of Zn (µM). After 48h, supernatant was collected for a LDH release assay **(C)** and images were acquired **(D). C)** Triton-X 100 (0.1% for 24 h) served as a positive control for macrophage damage. Results are mean ± SD of 2-3 technical replicates, representative of 2 independent biological replicates. Each dot indicates a technical replicate.

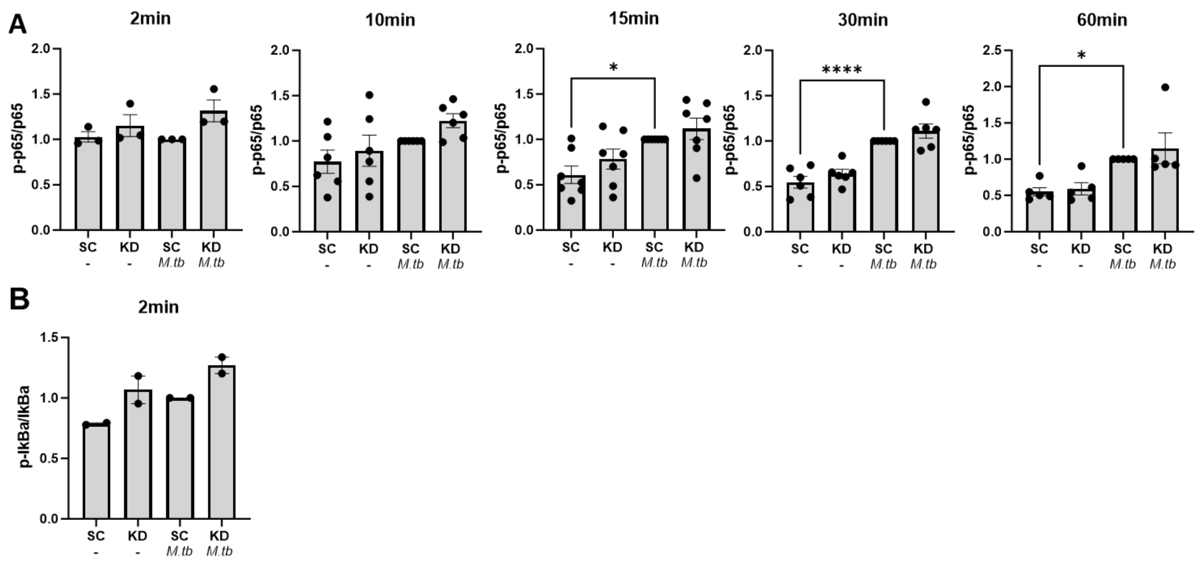

Fig S3. ZIP8 dampens NFκB activation during *M.tb* infection of macrophages. MDMs transfected with scrambled control (sc) or ZIP8 (KD) siRNA were infected with *M.tb* (MOI 10) and protein lysates collected at the indicated times. Protein levels of p-p65, p65, p-IκBα, IκBα were assessed by Western blot, then quantified. **A)** Results are mean ± SEM, n=3 (2 min), 5 (60 min), 6 (10,30 min), 7 independent biological replicates (15 min). **B)** Results are mean ± SEM, n=2 independent biological replicates. **A,B)** Each dot indicates an independent biological replicate. One-way ANOVA with Dunnett's post-test, * $p < 0.05$, **** $p < 0.0001$.

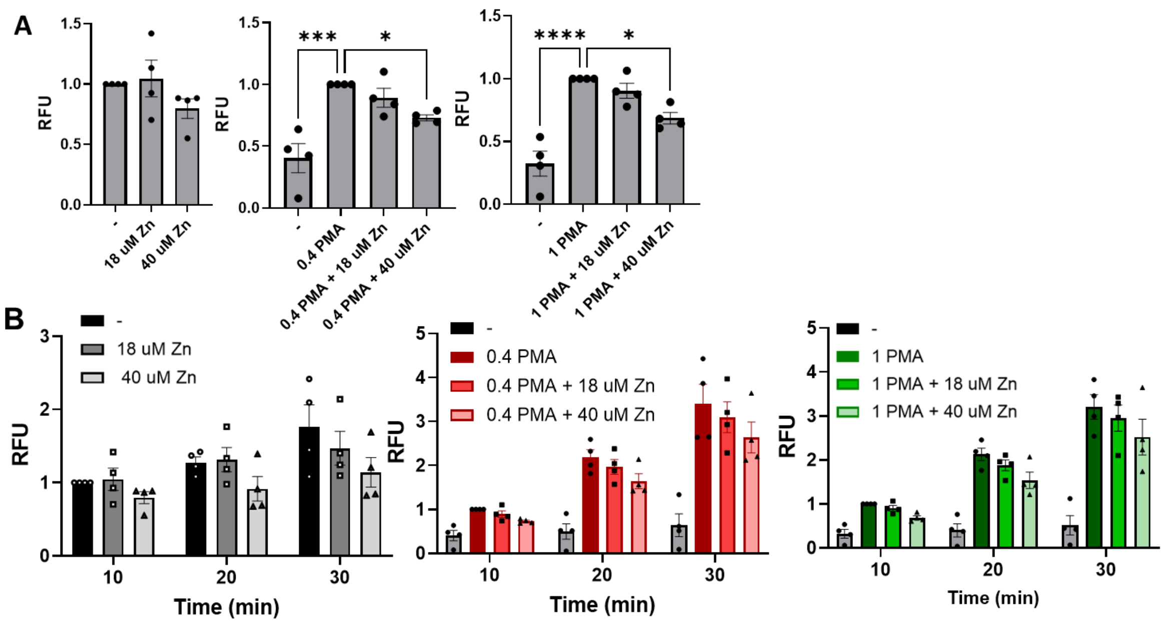

Fig S4. Zn dampens basal and PMA-induced ROS of macrophages. MDMs were cultured with 30 µM DCF in DPBS-HHG for 30 min at 37°C. Cells were treated with Zn at the indicated concentrations or PMA (0.4, 1 µg/ml), or medium-only control and cultured at 37°C. Fluorescence of DCF, indicative of ROS, was measured for 10min **(A)** or 10- 30min **(B)** at the indicated times. Results are relative fluorescence units (RFU), relative to no Zn controls at 10min, mean ± SEM of four independent biological replicates with two technical replicates each, each point indicates an independent biological replicate, one-way ANOVA with Dunnett's post-test, * $p < 0.05$, *** $p < 0.001$, **** $p < 0.0001$.

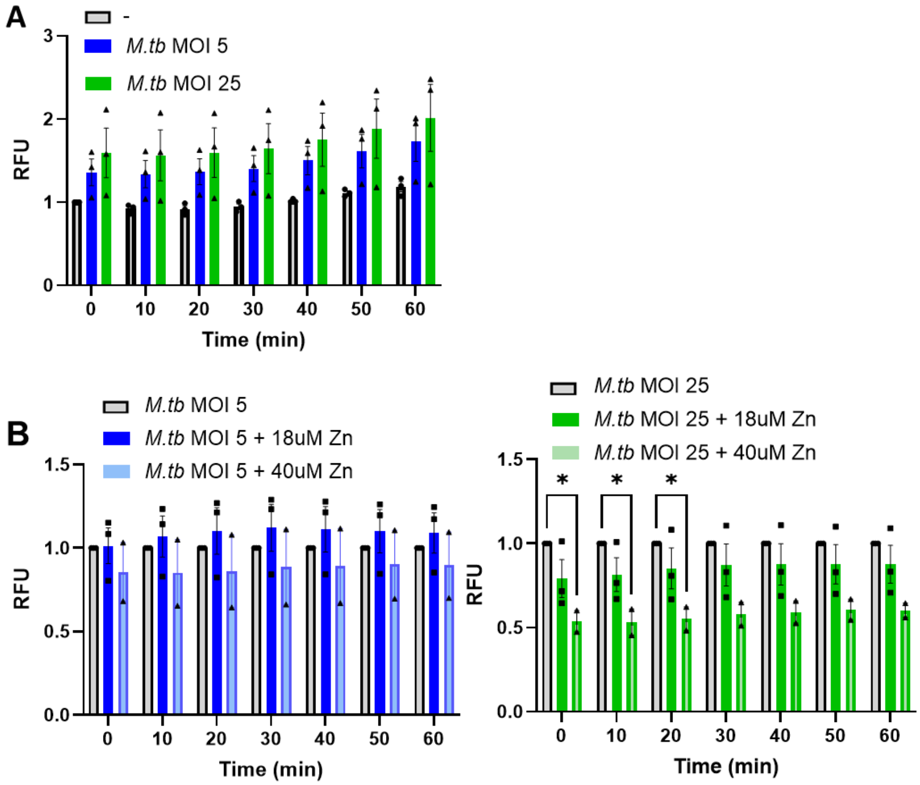

Fig S5. Zn dampens *M.tb*-induced macrophage ROS. MDMs were cultured with 30 μM DCF in DPBS-HHG for 30 min at 37°C. To synchronize phagocytosis, MDMs were cooled to 4°C for 10 min, then Zn and *M.tb* were added. Cells were centrifuged at 350 g for 10 min, then cultured at 37°C. Fluorescence of DCF, indicative of ROS, was measured at the indicated times. **A)** Results are RFU, relative to unstimulated controls at Time 0. **B)** Results are RFU, relative to *M.tb* infected control without Zn addition at Time 0. **A-B)** Results are mean ± SEM of two (40 μM Zn) to three (0, 18 μM Zn) independent experiments with two technical replicates, each point indicates an independent biological replicate, two-way ANOVA with Dunnett's post-test * $p < 0.05$.

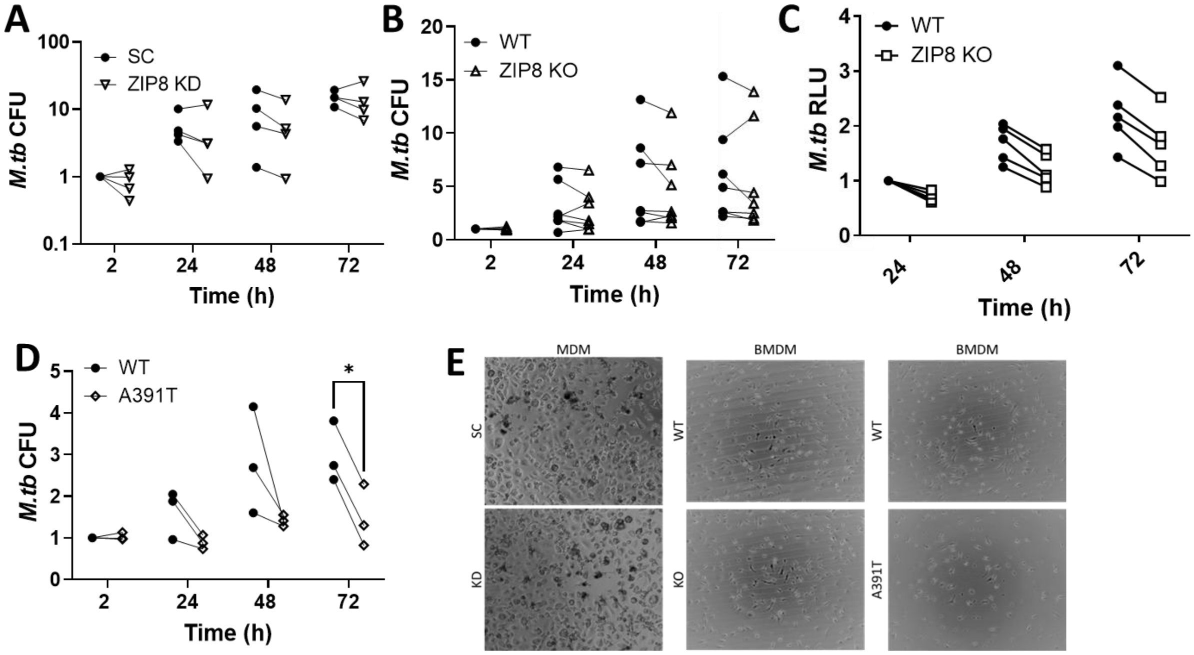

Fig S6. ZIP8 is important for *M.tb* growth in macrophages. A) MDMs were transfected with scrambled control (sc) or ZIP8 specific (KD) siRNA then infected with *M.tb* (MOI 5). At the indicated times post infection cells were lysed and CFU enumerated. n = 4 independent biological replicates. **B,C)** Bone marrow-derived macrophages (BMDMs) were isolated from WT and ZIP8 knockout (KO) mice. Macrophages were infected with *M.tb* **(B**, n=6**)** or *M.tb*-lux **(C**, n=5**)** at MOI 5. At the indicated times post infection cells were lysed and CFU enumerated **(B)** or relative luminescence units (RLU) assessed **(C)**. **D)** BMDMs were isolated from WT or A391T hypomorphic variant mice. Macrophages were infected with *M.tb* (MOI 5). At the indicated times post infection cells were lysed and CFU enumerated, n=3. **A-D)** Results are mean of at least three independent experiments with three technical replicates each, each point indicates one experiment. Two-way ANOVA with Sidak's post-test, * $p < 0.05$. **E)** Representative images of macrophages 72 h after *M.tb* infection.

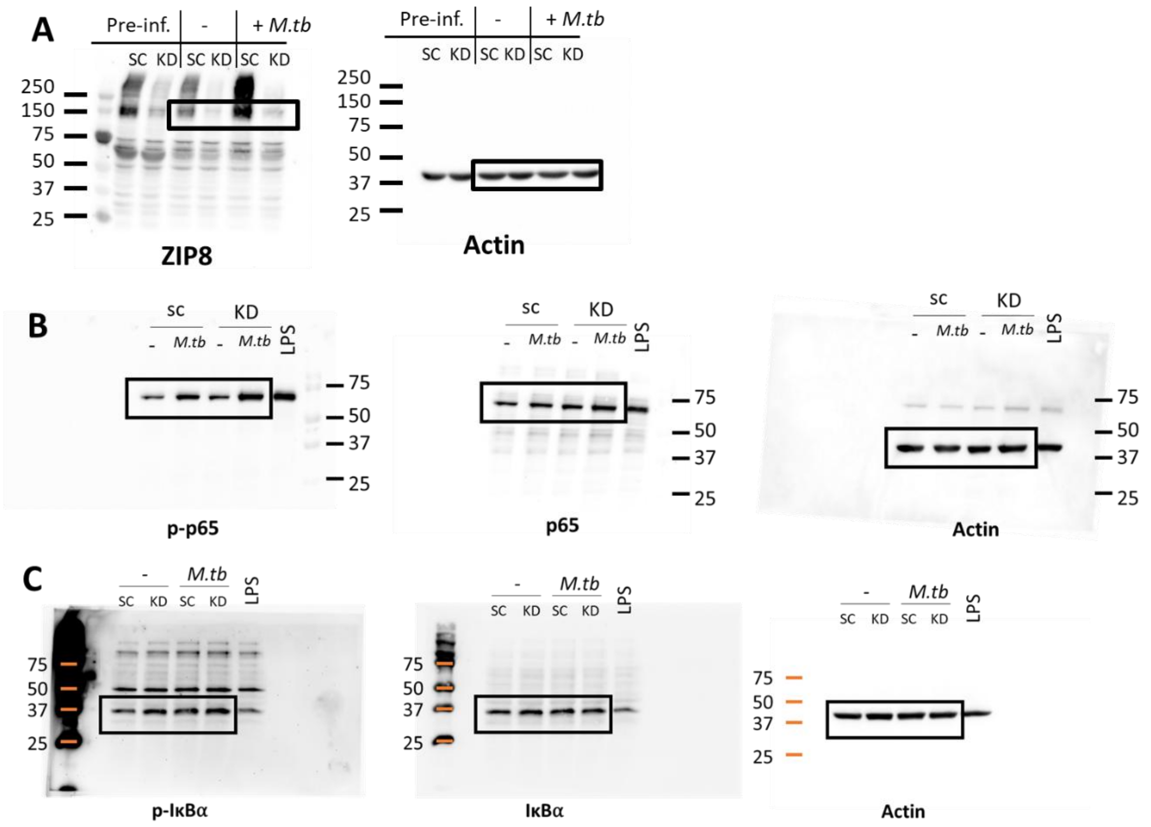

Fig S7. Uncropped Western Blots. Representative uncropped blots of those shown in Fig 2A **(A)**, Fig 3D **(B)**, and Fig 3E **(C)**. **B,C)** LPS was used as a positive control.
